# Beyond the mouse: 3R-guided alternative animal models transforming cancer research

**DOI:** 10.1186/s12943-026-02601-0

**Published:** 2026-03-26

**Authors:** Tobias Faehling, Tobias Reiff, Björn Schumacher, Richard Arndt, James F. Amatruda, Thomas G. P. Grünewald, Florencia Cidre-Aranaz

**Affiliations:** 1 Hopp Children`s Cancer Center Heidelberg (KiTZ), Heidelberg, Germany; 2 National Center for Tumor Diseases (NCT), NCT Heidelberg, a partnership between DKFZ and Heidelberg University Hospital, Heidelberg, Germany; 3 Division of Translational Pediatric Sarcoma Research, German Cancer Research Center (DKFZ), German Cancer Consortium (DKTK), Heidelberg, Germany; 4 Department of Paediatric Oncology, Haematology, Oncology and Immunology, Heidelberg University Hospital, Heidelberg, Germany; 5 Institute of Genetics Heinrich-Heine‐University Düsseldorf, Düsseldorf, Germany; 6 Institute for Genome Stability in Aging and Disease, University and University Hospital of Cologne, Cologne, Germany; 7 Cologne Excellence Cluster for Stress Responses in Ageing-Associated Diseases (CECAD), University and University Hospital Cologne, Cologne, Germany; 8 Faculty of Medicine, Heidelberg University, Heidelberg, Germany; 9 Cancer and Blood Disease Institute, Division of Hematology-Oncology, Children’s Hospital, Los Angeles, CA USA; 10 Department of Pediatrics, Keck School of Medicine, University of Southern California, Los Angeles, CA USA; 11 Institute of Pathology, Heidelberg University Hospital, Heidelberg, Germany

**Keywords:** 3R principle, Cancer modelling, CAM, C. elegans, Drosophila, Zebrafish

## Abstract

Comprehensively understanding cancer biology requires complex model systems, typically animal models, to replicate tumor behavior, microenvironment interactions, and cell migration/metastatic processes. Traditionally, genetically engineered and xenograft mouse models have been the cornerstone of cancer research. In recent years, the 3R principle—Replacement, Reduction, and Refinement—has reshaped ethical cancer research by promoting alternative animal models. Here we critically examine *in vitro* and *in silico* as well as four 3R-compliant animal models — the chicken chorioallantoic membrane (CAM) model, *Danio rerio*, *Drosophila melanogaster*, and *Caenorhabditis elegans* — and assess their advantages, limitations, and translational relevance for cancer research. Special emphasis is placed on practical considerations to inform optimal decision-making, including reproducibility and model-specific advantages and challenges. This review aims to support researchers in selecting ethical and effective preclinical models to advance cancer research.

## Introduction and 3R principle

While all animal experimental approaches must be conducted under strict ethical and legal frameworks, and with predefined humane endpoints, the systematic study of animal experimental techniques further led to the proposal of the 3R principle — Replacement, Reduction, and Refinement — in 1959: Replacement, as a proposal to use alternative models instead of animals whenever possible; Reduction, as an effort to minimize the number of animals used per experiment and the overall number of experiments per study; and Refinement, with the purpose of decreasing animal distress and improving their welfare [1]. These principles were introduced to increase the reliability of experiments by reducing the unwanted and uncontrolled stimuli introduced by distress and by concern for the animal’s wellbeing itself. More recently, increasing awareness of the broader consequences of animal experimentation—including public scrutiny, ethical responsibility, and the impact of animal mistreatment on the well-being of scientific staff—has contributed to the tightening of regulatory requirements [2–4]. In this context, the development of a 3R framework and incorporating 3R-compliant models has gained substantial importance in the field of biomedical research [5, 6].

The 3R principle can be readily applied by combining *in vitro* (two-dimensional (2D) and three-dimensional (3D)) and *in silico* modeling. Nevertheless, while these methods are becoming increasingly sophisticated, they still cannot fully recapitulate complex tumor-stroma interactions, immune responses, and metastatic processes, among others [7, 8]. Thus, animal models—usually rodents—remain a cornerstone of basic, physiological, and translational cancer research [9].

In recent years, alternative animal models have experienced a renaissance as viable substitutes or complements to rodent models, since the former are generally perceived as less sentient compared to fully developed rodents [10–12]. Consequently, international regulations consistently exempt invertebrate models (e.g., *Caenorhabditis elegans*, *Drosophila melanogaster*) from animal-use legislation, while vertebrate models like zebrafish and avian embryos become legally protected only beyond certain developmental stages. In Canada, Australia, the UK, and the EU, laws or guidelines protect vertebrates depending on developmental stages (e.g., from the point of independent feeding) and cover avian species only after hatching or late-term incubation, leaving earlier embryos unregulated [13–17]. Similarly, Japan’s self-regulation framework omits fish (such as *Danio rerio*,* known as ‘*zebrafish’) and invertebrates, focusing on mammals, birds, and reptiles, while China’s regulations apply 3Rs-based oversight only to traditional vertebrate laboratory animals [18].

In this context, the most historical non-rodent model discussed in this review is the chick embryo chorioallantoic membrane (CAM) model, with first xenotransplantation studies done in 1911 [19]. In the 1980s, modeling cancer cell invasion and metastasis was first introduced by the pioneering work of L. Ossowski [20, 21]. Similarly, *D. melanogaster* (fruit fly) emerged as a model organism for genetic research, with the first hereditary tumor being described in 1919 [22]. Subsequent landmark discoveries in developmental genetics, most notably the identification of patterning genes recognized by the 1995 Nobel Prize, fundamentally advanced the field [23–25]. These insights laid the foundation for genetically engineering human tumors in *Drosophila* by manipulating oncogenes and tumor suppressors from the 1980s onward [23–27]. Zebrafish emerged as a cancer model in the 1980s, providing a transparent system for live imaging and high-throughput drug screening [10, 28–31]. Further, cancer research on *C. elegans* began with the discovery of apoptosis-regulating genes in the 1970s and 1980s, eventually leading to the Nobel Prize in 2002 [32, 33]. Thus, this review will focus on these frequently used and flexible alternative animal models: avian embryos, fruit fly, zebrafish, and the nematode worm *C. elegans* [11, 34, 35].

Advances in imaging, genetic manipulation, and microenvironmental engineering have enhanced the reliability and applicability of these models [36]. Moreover, the advent of personalized medicine—mostly relying on cell culture or murine PDXs—has further driven the development of patient-derived models that better mimic human cancers while aligning with the 3R principles [37]. This renewed interest reflects a broader paradigm shift in preclinical research, where integrating alternative model organisms with traditional approaches may enhance the success of basic and translational cancer studies.

This review presents 3R-compliant alternative models and comparatively discuss their strengths and limitations, facilitating the selection of ethical and effective preclinical models to advance cancer research.

## Non-animal models

### *In silico* modeling of cancer

*In silico* modeling of cellular behavior and cancer progression offers the possibility to conduct a high number of experiments in parallel, elucidating many potential pathophysiological and pharmacological parameters. Aggregating data from different patients or even cancer types to simulate tumor growth, invasion, angiogenesis, and immune interactions integrated with clinical data may aid in refining hypotheses and optimizing therapeutic strategies, thus aligning with the Reduction principle [38, 39].

Structural bioinformatics and molecular dynamics simulations enable detailed modeling of protein folding, stability, and interactions, supporting antibody discovery and the engineering of cellular therapies by predicting antigen-binding and receptor-ligand dynamics [40]. The integration of clinical datasets into artificial intelligence (AI)-driven modeling frameworks further advances the development of personalized medicine, allowing prediction of patient-specific treatment responses, resistance mechanisms, and disease progression [41, 42].

Although ongoing improvements in machine learning, data integration, and biophysical modeling have markedly enhanced *in silico* models, it should be noted that they represent a simplification of the biological complexity of cells and organisms, and their predictions still require experimental validation [41, 42].

### Conventional 2D cell culture

Preclinical oncology has significantly advanced through the use of established tumor cell lines, especially due to their ease of handling, low cost, and broad availability [43]. Culturing cell lines in 2D enables the controlled investigation of drug response, proliferation, migration, and phenotypic plasticity under standardized conditions [44, 45]. However, 2D cultures cannot replicate the complex 3D architecture, biochemical gradients, or cell-matrix interactions present in tumors. In addition, their potential clonal evolution may result in limitations in experimental reproducibility [46, 47]. As a result, their predictive value for *in vivo* tumor behavior and therapeutic response studies is limited, highlighting the need for more physiologically relevant models in translational cancer research [48–50].

### Physiological 3D or organoid cell culture

3D cell culture technologies better mimic the cell states *in vivo* by enabling physiological cell-cell and cell-matrix interactions [51, 52]. Tumor spheroids and patient-derived organoids preserve key features such as tumor heterogeneity, tissue-specific architecture, and nutrient and oxygen gradients, supporting more accurate modeling of growth dynamics, therapy resistance, and metastatic behavior [52–54]. In addition, organoids retain the molecular profiles of primary tumors, making them valuable platforms for personalized medicine and biomarker discovery [55]. Finally, organ-on-a-chip systems combine 3D cultures with microfluidics to recreate dynamic perfusion, mechanical forces, and complex tissue organization, further allowing real-time monitoring of cancer cell invasion, extravasation, and interactions with stromal and immune cells under controlled conditions [56, 57].

However, despite these advances over two-dimensional cultures, 3D *in vitro* systems remain fundamentally reductionist. They lack integration in systemic cues and fail to recapitulate critical features of full organisms, including tissue-tissue interactions, functional blood and lymphatic circulation, physiological immune composition, endocrine and metabolic regulation, and dynamic chemical and hormonal fluctuations and gradients related to nutrition or circadian rhythms [44, 58–60]. Moreover, they often rely on exogenous, researcher-defined supplementation (e.g., growth factors and matrices), which can substantially shape cellular states and thereby influence tumor cell physiology and therapy responses [61]. While some of these parameters can be partially modeled using organ-on-a-chip or microfluidic platforms, they are typically addressed in isolation, at high technical complexity and cost, and still rely on artificial, plastic-based environments. These limitations are discussed in detail in several comprehensive reviews of 3D cancer models and organoid systems, which underscore their value as intermediate experimental platforms rather than full surrogates of *in vivo* biology [51, 62, 63].

## Chorioallantoic membrane model (CAM)

### General experimental approach and alternative setups

The CAM model offers a versatile and accessible *in vivo* platform for investigating human tumor biology, metastasis, and therapeutic responses. Since several dozen biological replicates can be feasibly performed in parallel in CAM studies every three weeks, this model is also amenable to studying a high number of biological variables [64].

During early embryonic development, the chorion and allantois fuse to form the CAM, which progressively expands to cover most of the eggshell surface [65]. Due to its important role in gas exchange and metabolism, the CAM develops a dense and highly organized vascular network, while its extraembryonal localization results in a lack of innervation [65–67]. These characteristics provide a permissive, well-perfused environment for tumor growth and manipulion.at

**Fig. 1:**
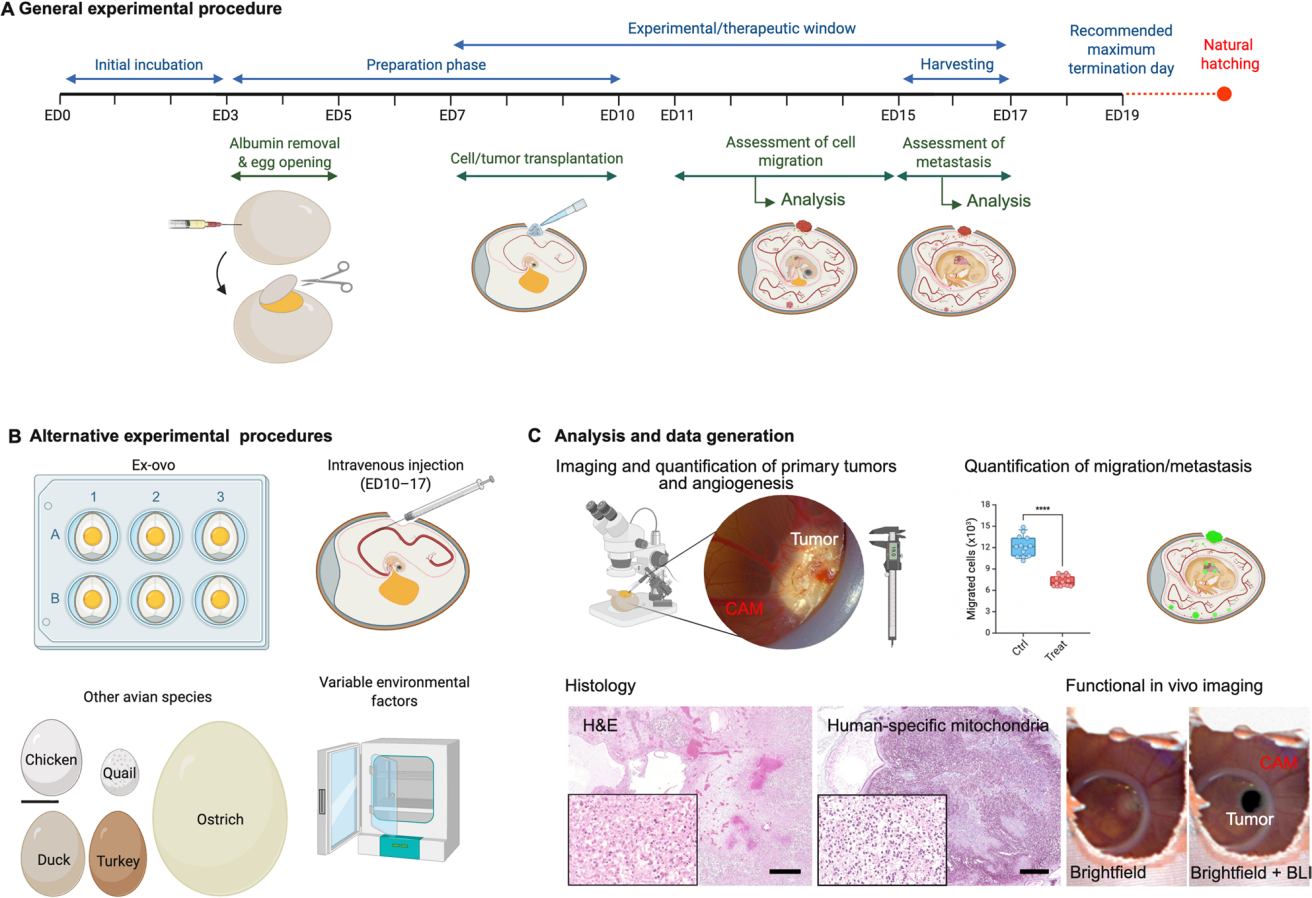
Overview of avian CAM model. **A**. Timeline for a general experimental procedure. ED: embryonic day. **B**. Schematic representation of alternative experimental procedures, including *ex ovo* assays, intravenous injection, employment of other avian species (chicken egg displayed for reference, scale bar = 35 mm), and different environmental factors that may be controllably modified. **C**. Analysis and data generation. Top, left: Stereomicroscopy with sample image of a CAM-grown Ewing sarcoma primary tumor where the vasculature can be quantified. Top, right: Quantification of migrated cells by qPCR analysis of human-specific Alu-sequences (left). Schematic view of fluorescently marked cells (GFP, green) disseminating in the embryo (right). Bottom, left: Histology of a CAM-grown desmoplastic small round cell tumor stained with H&E (left) and human mitochondria antibody (right). Bottom, right: Functional in vivo imaging of luciferase-transfected cells of a CAM-grown Ewing sarcoma (left: brightfield, right: overlay with bioluminescence imaging (BLI) signal). Created in BioRender (https://BioRender.com)

Typically, CAM experiments comprise sequential incubation, transplantation, and analysis phases, during which the embryos are maintained under controlled conditions, usually entailing incubation at 37.5 °C and 70–90% relative humidity [pic^66^, ^67^]. After early embryonic development, the CAM is rendered accessible and allowed to mature to a stage permissive for experimental manipulation, including tumor transplantation and co-culture with stromal components [36, 64, 67–70] (Fig. 1A). Human tumor material, including patient-derived fragments or cell suspensions, can be transplanted onto the CAM, optionally in combination with human stromal components such as fibroblasts, endothelial cells, or immune cells to approximate features of the tumor microenvironment (TME) [71]. Using established protocols, high tumor engraftment rates of ≥ 80% can be achieved [36, 64, 68, 72, 73]. Following inoculation, tumor growth can be monitored (see chapter ‘Analysis and experimental readouts’). Finally, tumors are harvested along with the surrounding CAM tissue for subsequent histological, molecular, or functional analyses [69, 74] (Fig. 1A). A collection of detailed step-by-step protocols on each of the aforementioned interventions is already established [36, 64, 67–70].

Several protocol adaptations of the CAM model have been developed to allow for assessment of additional features. These adaptations range from maintaining the eggs in hypoxic conditions to mimicking pathophysiological processes [75] (Fig. 1B). Additionally, for metastasis studies, tumor cells can be recovered from distant sites at the embryo or tracked to investigate dissemination and secondary lesion formation [67]. Moreover, to directly assess the late steps of the metastatic cascade, tumor cells can be injected into CAM vessels so extravasation and metastasis formation can be studied [76] (Fig. 1B). Alternative avian species, such as quail and duck, offer differing egg sizes and developmental rates, either prolonging the experimental window or improving throughput [77, 78] (Fig. 1B). Interestingly, due to their size, ostrich eggs are compatible with clinical radiological and radiotherapeutical devices and can provide more time and space for experimental manipulation [79].

To enhance both physical and optical access to the embryos, *ex ovo* culture systems have been developed, where embryos are transferred from their shells onto sterile supports at ED 3^Ref.77^. It should be noted that both ostrich and *ex ovo* models enable the seeding of multiple tumors within a single embryo, facilitating direct comparison of experimental and control groups in a shared microenvironment [78] (Fig. 1B).

### Analysis and experiment readouts

Complying with the 3R principle mandates an efficient use of the avian embryos, which requires accurate tracking and evaluation of tumor characteristics on the CAM [1, 2]. Several hallmarks of cancer, including proliferation, invasion, vascularization, and therapy resistance, can be assessed using a combination of *in ovo* imaging, histopathology, and molecular approaches in the CAM model [80–83].

Cell proliferation and viability can be longitudinally tracked by stereomicroscopy, bioluminescence (BLI), and fluorescence imaging (Fig. 1C). While stereomicroscopy captures the morphology of the transplant and the surrounding CAM, BLI intensity reflects tumor size and viability [80]. Fluorescent proteins or tracking dyes can be employed to achieve stronger signals than BLI or functional read-outs [84, 85]. Importantly, even the development of micrometastases can be detected using fluorescent microscopy in *ex ovo* experiments [86] (Fig. 1C).

Angiogenesis can be quantified by injecting tracers into CAM vessels, which enables analyses of vessel density, morphology, and functional characteristics [87]. To improve reproducibility, deep learning tools, such as the IKOSA platform, can be employed for automatic angiogenesis quantification [87]. Alternatively, laser speckle contrast imaging (LSCI) can measure perfusion dynamics within angiogenic networks [88] (Fig. 1C).

Migration and metastasis can be either analyzed locally by quantifying the invasion of tumor cells in the CAM or systemically by detecting cell dissemination to distant parts of the embryo. Additionally to fluorescence, human-specific Alu PCR can be employed to enable highly sensitive detection of micrometastases in the distant CAM with a detection range of even < 100 cancer cells [67, 89–91] (Fig. 1C).

It should be noted that the CAM is amenable to the use to radiological approaches that mirror those in clinical practice. For instance, similar to BLI, Positron emission tomography (PET) imaging enables viability tracking due to the metabolism-dependent uptake of radiotracers. Moreover, tracers targeting different tumor entities and biological processes are available, and their pharmacodynamics in CAM are comparable to those of murine xenografts [89, 92]. In addition, using ultrasound, tumor growth can be exactly determined and longitudinal analysis of angiogenesis is possible [93] (Fig. 1C).

Finally, several hallmarks of cancer can be assessed by histopathological and deep molecular profiling. Hematoxylin and eosin (H&E) staining, illustrated in Fig. 1C, reveals tumor architecture, stromal invasion, and vascular density. Importantly, CAM xenografts have shown to closely mimic the histomorphology found in patient tumors [64, 68]. Immunohistochemistry (IHC) using universal or tumor-specific human markers vs. avian-specific stainings can be used to distinguish grafted tumors from host tissues, while functional markers may be employed to characterize tumor states [64, 68, 80, 94].

Together, the versatility of readout strategies establishes the CAM model as a powerful and reproducible platform for translational oncology research.

### Therapy modalities

The CAM model is compatible with a wide array of therapy strategies commonly used in patients. CAM xenografts enable medium-throughput screening of anticancer drugs, including their effects on tumor growth, angiogenesis, and metastatic dissemination [69, 95, 96]. In terms of therapeutic administration, drugs can either be applied topically, injected into the albumen, or intravenously, and different delivery strategies such as antibodies, nucleic acids, nanoparticles, or proteoliposomes are possible [64, 96–100]. Interestingly, avian embryos exhibit a high tolerance to radiation in clinical doses, making them a feasible model for radiotherapy treatment assessment [101].

Due to the relatively high throughput of CAM experiments, treatment schemes and pharmacological combinations can be readily optimized [102]. In addition, the multiplicity of available drug administration strategies allows for genetic manipulation and gene therapy to be employed, including the use of nucleic acids and tumor-targeting viruses. Indeed, Huang and colleagues showed that overexpression of a tumor-suppressor microRNA in a non-small cell lung cancer xenograft inhibited tumor growth and angiogenesis in the CAM model [103]. Additionally, Krutzke and colleagues used human adenovirus type 5 (HAdV-5) to assess virus–host interactions, biodistribution patterns, and tumor-targeting profiles in the CAM model [104]. However, the embryonic nature of the host imposes important constraints on dosing of many chemotherapeutic or embryotoxic agents, as systemic exposure can rapidly lead to malformations or lethality at concentrations below human‑equivalent levels [68, 105]. Consequently, several cytotoxic drugs must be tested at empirically down‑titrated doses that prioritize embryo viability over pharmacokinetic matching, limiting the extent to which CAM readouts can be interpreted as quantitatively human‑relevant for such compounds [99, 106].

Furthermore, light-based modalities are likewise well suited to the CAM. Kerkhoff and colleagues used 5-ALA-mediated photodynamic therapy in a sarcoma model to disrupt tumor vasculature and induce tumor regression [107]. Owing to the high vascularization of tumor transplants, the CAM model can be used to evaluate angiogenesis inhibitors and vascular disrupting agents. Of note, the ability to conduct live microscopy allows real-time monitoring of angiogenesis, endothelial breakdown and localized hemorrhage [106, 108].

Although the chick embryo has an immature immune system, where no immune cells are present until ED 10–11, it has recently been described that the CAM model can still be utilized for immunotherapy research [109–112]. Indeed, human PD-1/PD-L1 checkpoint blockade in tumor-bearing CAMs significantly slowed tumor growth, and co-implantation of human immune cells or chimeric antigen T-cells with tumors could recreate human-like immune interactions *in ovo* [95, 111, 112].

Overall, the CAM model is an excellent resource to robustly test and combine a wide array of anti-cancer treatment strategies and can aid in bridging the transition from *in vitro* experiments to rodents and ultimately patients.

### Patient-derived tumor models

Patient-derived xenograft (PDX) models are important tools in oncology research, accurately reflecting tumor heterogeneity while faithfully recapitulating the features of its TME. Maintaining and propagating PDXs on the CAM using the standard methodology is straightforward (s. ‘General experimental approach’), with high tumor take rates across diverse cancer types [66, 113]. In fact, Charbonneau and colleagues showed that engraftment rates of > 95% could be achieved for glial tumors in the CAM model, whereas only 39–69% were reported in murine xenografts [113]. CAM-derived xenografts closely resemble the original tumors, preserving key histological features and intratumoral heterogeneity [37, 113]. The CAM provides rapid vascularization of the grafts, which enables the formation of micrometastases and local invasion – features that correlate with the original tumor’s aggressiveness [37]. Indeed, the CAM model has even been used to generate PDXs from circulating tumor cells (CTCs), including circulating cancer stem cells (cCSCs), while forming tumors that retain phenotypic and molecular features of the primary malignancy [114–116]. This approach provides insights into metastatic potential and supports drug testing in a rapid assay, representing a valuable tool for individualized therapy approaches [114–117].

The low turnaround time is especially relevant for the application in personalized medicine, where drug efficacy and treatment response can be screened to optimize therapy [113, 117]. Consequently, Barnabas and colleagues utilized chick PDXs for proteomic screening and drug testing, thereby optimizing the treatment strategy for a patient with progressive disease [117].

Thus, the CAM-PDX model is increasingly recognized as a practical complement to murine xenografts and *in vitro* long-term patient-derived cultures, particularly for personalized oncology applications requiring time-efficient, biologically relevant tumor modeling.

### Advantages and limitations

The CAM model offers several key advantages compared to other cancer models: (a) Its natural immunodeficiency allows xenografting of human tumor cells without immune rejection; (b) Tumors develop within 5–10 days, enabling rapid and medium-throughput functional and drug screening; (c) It supports histologically and molecularly representative tumors, preserving stromal and vascular components; (d) Its dense vasculature facilitates real-time imaging of tumor angiogenesis, metastasis, and cell behavior; (e) Its immediate optical accessibility facilitates longitudinal imaging studies (f) It accommodates a wide range of tumor sample types, including cell lines, patient-derived xenografts (PDXs), and circulating tumor cells (CTCs); (g) It requires a relatively low initial investment in infrastructure, as egg incubators are often inexpensive; (h) The involvement of ethical committees is normally not required; and (i) Topical and systemic drug administration is straightforward. Overall, this general accessibility makes it well-suited for early-phase *in vivo* testing, particularly in settings with limited resources, or for the time-sensitive personalized medicine.

Nonetheless, several limitations must be considered: (a) The short experimental window prevents assessment of long-term features such as tumor remission, recurrence, or chronic drug effects; (b) Oral administration of drugs or pre-drugs is not feasible, and compounds must be solubilized for injection, posing challenges for poorly soluble agents; (c) As in any whole-organism model, systemic dosing is constrained by tolerability and embryo viability, which enforces physiologically relevant exposures but may limit escalation for poorly tolerated compounds or solvent systems; (d) Even though *in vivo* imaging is possible, 3D imaging of tumor growth is limited by the eggshell opacity; (e) Environmental factors—such as oxygen tension, pH, temperature, and humidity—should be carefully controlled to prevent confounding effects; and (f) Similar to other animal models, inter-species immunological and/or metabolic variability may limit the translational potential of individual results.

In sum, the CAM model is best used for short-term, cost-effective, medium-throughput *in vivo* studies focused on tumor engraftment, angiogenesis, metastasis, or drug efficacy. It is a powerful screening tool that bridges the gap between *in vitro* experiments and murine models. However, its biological and methodological constraints necessitate concise follow-up studies in mammals to ensure translational reliability.

## Zebrafish (*Danio rerio*)

### General experimental approach and alternative setups

Growing out of the pioneering work of George Streisinger and colleagues beginning in the 1980s [118, 119], zebrafish became widely adopted as an experimental model organism with the publication of the first large-scale genetic screens in 1996 [31, 120]. From this early work, the advantages of the zebrafish system—including powerful genetics, large clutch size, and rapid development of transparent embryos external to the mother—were already evident.

Since a pair of zebrafish can produce several hundred fertilized embryos from a single mating, and nucleic acids or proteins can easily be injected into embryos at the single-cell stage, they are amenable to high-throughput genetical modeling. Of note, a zebrafish ortholog is known for 70% of human genes (85% of disease-related genes [121]), and the organ structure and physiology of fish is similar to other vertebrates, including hematopoiesis, liver synthetic and metabolic function, vascular and lymphatic systems and both innate and adaptive immunity [122–124]. Early genetically-engineered zebrafish models of T-cell leukemia [125] and melanoma [126] established the utility of the zebrafish model for building animal models that recapitulated key features of human cancers. Further development of technologies such as transposon-based transgenesis and mutagenesis [127, 128] and implementation of conditional gene expression strategies [129–131] enabled rapid expansion of the spectrum of cancers for which models were available, including neuroblastoma [132], sarcomas [133–136], hepatocellular cancer [137], brain tumors [138, 139] and others [29, 140]. These models have been leveraged for functional genomics [141, 142] and for insight into the cell lineage of origin of cancers [141, 143]. Hence, in a typical experiment, a tumor model is generated by mosaic, conditional, and/or tissue-specific expression of an oncogene or loss of a tumor suppressor [144].

Zebrafish are sensitive to carcinogens and can develop a wide range of malignancies [145, 146]. Consequently, methods have been developed to expose fish to carcinogens as well as antineoplastics, small molecules, cellular therapies, radiation, and other therapies (s. ‘Therapy modalities’).

In addition to genetic models, another major use of zebrafish is as a platform for xenograft studies. Pioneering work by Nicoli and Presta demonstrated that human tumor cells implanted into zebrafish embryos could stimulate neovascularization from host vessels and that anti-angiogenic agents could suppress this response [147]. Zebrafish embryos rapidly develop a functional vascular and lymphatic circulatory system over the first few days of life [148–150] and the transparent embryos and larvae support high-content imaging [28, 151, 152]. Transgenic zebrafish embryos expressing fluorescent reporters in vasculature also facilitate studies of tumor-related angiogenesis and the effects of anti-angiogenic therapies [153–156]. Leveraging immunodeficient zebrafish strains, xenograft experiments can also be performed in adult fish [157]. Mutagenized strains that lack pigmentation even at adult stages are also available [158]. Subsequently, the xenograft model has been adopted for a wide range of studies of cell- and patient-derived xenografts (s. ‘Patient-derived tumor models’).

### Analysis and experiment readouts

One of the big advantages of the zebrafish system is the optical transparency of embryonic and larval zebrafish, which allows detection and tracking of fluorescently labeled tumor cells. Fluorescent proteins such as eGFP can be incorporated into tumor models and xenografts, which facilitates high-resolution imaging at the single-cell level.

**Fig. 2:**
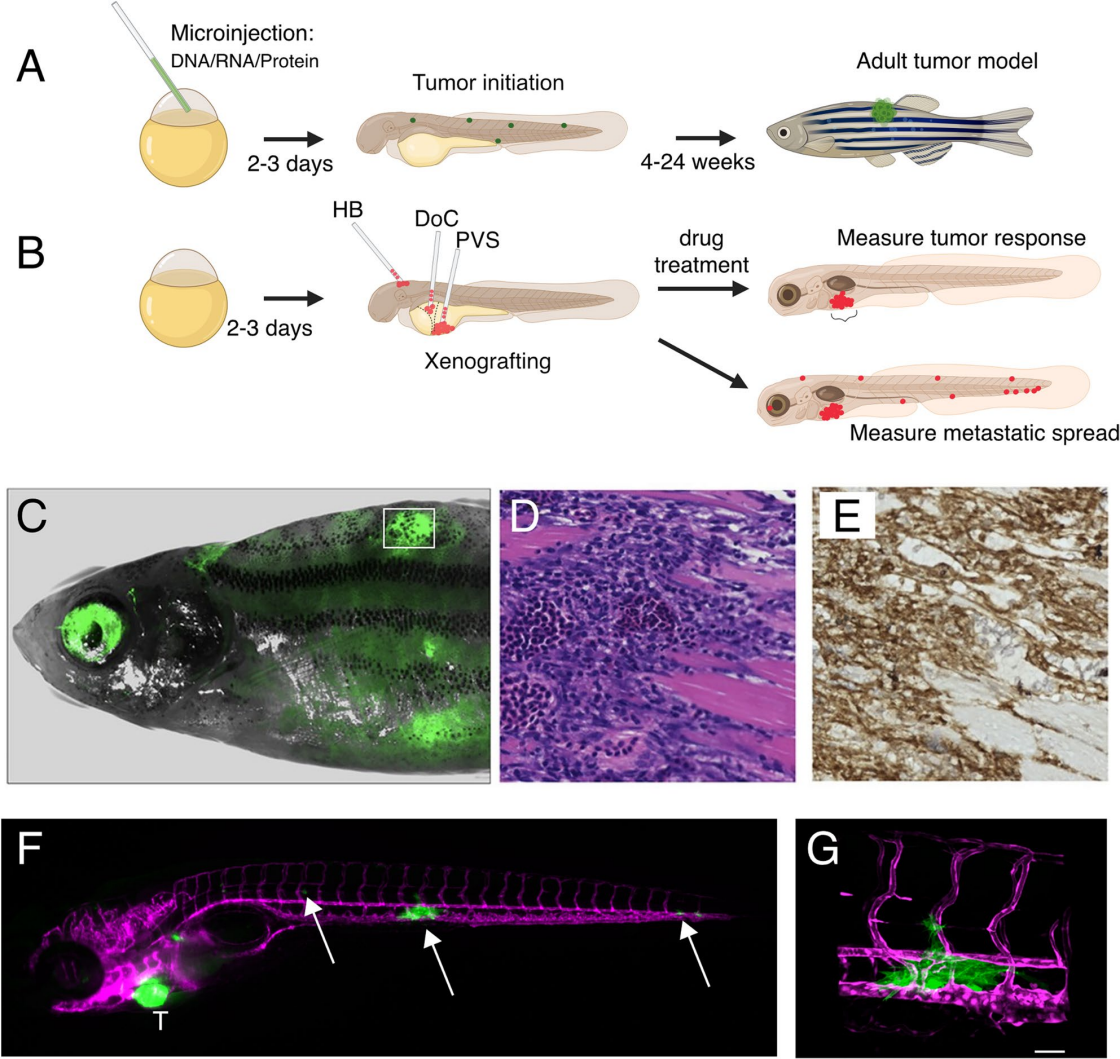
Overview of *D. rerio* zebrafish model. **A.** Generation of genetically engineered tumor models. Single cell-stage embryos are injected with DNA, RNA and/or protein to activate or repress cancer-related genes of interest. Inclusion of a fluorescent marker allows monitoring of the effects of genetic manipulations during early embryonic and larval stages. Over weeks to months, animals may develop tumors which can be further analyzed. **B**. Zebrafish xenograft system. Fluorescently labeled human cancer cells are implanted into larval stage zebrafish. Cells are implanted into the hindbrain ventricle (HB), the ventral perivitelline space (PVS), or via intravascular injection into the Duct of Cuvier (DoC). Xenografted embryos can be exposed to drugs such as small molecules to measure antitumor effects. Alternatively, the spread of tumor cells to distant sites can be used as a measure of metastatic potential. **C-E.** Analysis of tumor histology. **C**. 3-month-old zebrafish with mosaic expression of the human EWSR1::FLI1 fusion oncogene. Green fluorescence (white rectangle) marks tumor site. **D**. H&E stain of muscle-infiltrating tumor showing small round blue cell histology consistent with Ewing sarcoma. **E**. Immunohistochemical detection of CD99, a clinical marker of Ewing sarcoma. **F**. Xenograft of GFP-labeled human CHLA9 Ewing sarcoma cells into kdrl: DsRed transgenic zebrafish larva. Magenta color marks vasculature. T: primary tumor implantation site. Arrows: metastatic cells. **G**. Confocal microscope imaging of CHLA9 cells extravasated into the space between the dorsal aorta (DA) and posterior cardinal vein (PCV). Scale bar: 50 microns. A and B created in BioRender (https://BioRender.com). C-E derived from Vasileva et al. eLife 2022. F and G courtesy of Ingrid Lekk, PhD

If a fluorescent label is incorporated into the transgenic construct, the area, volume, and intensity of tumor-specific fluorescence can be measured in multiple biological replicates to generate statistics for tumor growth rates, metastatic spread, and response to therapies (Fig. 2A, C). Live imaging allows serial measurements over time, beginning at the earliest stages of embryonic development, which can be complemented by endpoint assays on fixed tissue (either whole-mount preparations of embryos/larvae, or sections of adult fish tissue) including markers of cell proliferation, cell death, DNA damage, signaling pathway activity and others [159].

Histopathologic examination can be performed on cryosections or on sections of formalin-fixed, paraffin-embedded (FFPE) tissues, including hematoxylin & eosin (H&E) stains (Fig. 2D) and immunohistochemistry (Fig. 2E). A frequent limitation to immunostains of zebrafish cancer models is the lack of cross-reacting antibodies for specific zebrafish antigens. In situ hybridization to delineate expression of an mRNA of interest can provide a useful substitute. As with mammalian tissues, zebrafish tumors and surrounding non-tumor tissue can be subject to spatial transcriptomic techniques and multiplex immunofluorescence to generate a more detailed portrait of the tumor in its host environment [160].

Measurement of tumor growth can be performed by serial imaging using light microscopy, especially useful for pigmented superficial tumors such as melanomas. Alternative imaging modalities that have been applied include bioluminescence imaging, ultrasound, computed tomography, and magnetic resonance imaging [161–164]. Most frequently, incorporation of a fluorescent marker into the transgenic construct allows for live imaging by epifluorescence microscopy. The tumor mass can be quantified based on the area and/or intensity of the fluorescent signal. Fluorescence microscopy is also a mainstay in evaluating the fate of cells xenografted into larvae, most straightforwardly for measurement of tumor response to therapies [165]. The xenograft model also allows detection of cell spread outside the primary tumor as a measure of metastatic potential (Fig. 2B).

### Therapy modalities

As mentioned above, several routes are available for administration of therapies to zebrafish. Intraperitoneal injections can be easily performed in adult animals, and intravenous injection, widely available for rodent and larger animal models, is also possible in zebrafish. At the larval stage, capillary micropipettes can be used to inject small volumes into the duct of Cuvier, which drains venous blood into the sinus venosus of the heart. At the adult stage, intravascular access can be achieved via intracardiac, retroorbital, or intravenous injection [166]. Oral administration of drugs can occur via gavage into both larval and juvenile/adult fish or by incorporation of drugs into food [167–169]. Uniquely for aquatic organisms such as fish and frogs, drugs can be administered via waterborne exposure by simply adding compounds to the water. In the immersion approach, uptake occurs via a combination of absorption through the skin, gills, and by the animals swallowing drug-containing water. Though technically simple and especially useful for high-throughput screens performed on embryos [170, 171], poor solubility of drugs can impair the efficiency of aqueous administration.

Radiotherapy has also been successfully modeled in zebrafish, with studies showing tissue-specific and systemic effects of fractionated or single-dose irradiation in both larvae and adult fish [161, 172–174]. Zebrafish display dose-dependent DNA damage, cell death, and regeneration responses in tumor-bearing tissue, allowing the investigation of radiosensitizers or radioprotective agents [173].

Targeted therapies and biologics, including monoclonal antibodies, extracellular vesicles, lipid nanoparticles, and engineered immune cells (e.g., CAR-T cells), have been applied in zebrafish. These are typically administered via injection into embryos or adult fish and tracked via fluorescence or functional assays [175–178]. Studies evaluating the potential of immune checkpoint blockade experiments in zebrafish are ongoing [179].

Pharmacodynamic and pharmacokinetic (PD/PK) limitations persist, particularly regarding compound metabolism and clearance, as these are less well-characterized compared to rodent systems [171, 180]. However, the ease of serial imaging and biomarker quantification may act as surrogate parameters, which could partially compensate for these limitations, particularly in short-term efficacy studies.

Overall, zebrafish offer a scalable and versatile *in vivo* platform for evaluating drug efficacy, toxicity, mechanism of action, and delivery strategies—especially in the early stages of anti-cancer drug development.

### Patient-derived tumor models

One of the most rapidly growing uses of the zebrafish system is adaptation of the larval xenograft model for personalized medicine approaches, including studies aimed at predicting an individual tumor’s sensitivity to specific therapies or propensity for metastasis [181–183]. These zebrafish PDX (zPDX) models highlight the potential of fish to serve as a patient-specific avatar. A 3-day-old zebrafish has largely completed organogenesis and exhibits well-established vascular, lymphatic, and innate immune systems. Adaptive immunity is not present until the thymus develops at around 7–10 days of life. Thus, patient-derived tumor cells can be xenografted without the need for immunosuppression. Typical sites of implantation include the perivitelline space, the hindbrain ventricle, or directly into the circulation [165] (Fig. 2B).

Several zPDX approaches have been described, and the field lacks consensus on a single, gold-standard technique. To minimize phenotypic drift of patient-derived tumor cells and to avoid artifacts of *in vitro* culture, many zPDX studies rely on the use of lipophilic dyes to label human tumor cells. While dye labeling facilitates rapid labeling and visualization of the implantation, the dye may persist in dead cells or debris or be taken up by macrophages, potentially creating a false-positive signal. As an alternative, labeling of PDX cells with fluorescent reporter proteins or use of functional readouts (e.g., cell proliferation or cleaved Caspase 3) may be more reliable. On average, a maximum of several hundred tumor cells are implanted, which may limit the ability of a zPDX to truly represent intratumoral heterogeneity or to fully assess the impact of non-tumor cells such as macrophages and cancer-associated fibroblasts (though adult immunocompromised hosts can accommodate much larger numbers of cells [184]).

Despite these limitations, numerous studies have documented the utility and predictive power of the zPDX system. zPDX avatars have been used to test patient tumor-specific effects of radiation [174], chemotherapy, and small molecules [182, 185–188], to investigate effects of innate immune evasion [189], and to predict tumor metastatic propensity [190, 191]. zAvatars have been demonstrated to predict risk of progression in primary colorectal cancers [192], and a recent study of high-risk breast cancer compared results of the same therapy in patients and in matching zAvatars, finding 100% concordance between the zebrafish results and the patient’s clinical response [193]. The success of these approaches has prompted the design of a randomized clinical trial testing the utility of selecting treatment regimens based on the zAvatar assay [194].

### Advantages and limitations

The zebrafish system offers several notable advantages in cancer modeling: (a) Its optical transparency, especially in embryonic and larval stages, enables high-resolution, real-time imaging of tumor initiation, progression, and metastasis; (b) The high genetic and physiological overlap with humans enhances the translational value; (c) Genetic manipulation is rapid and cost-effective, allowing creation of transgenic models and CRISPR-based gene editing; (d) It supports both genetically engineered models and xenograft approaches, including patient-derived xenografts (zPDX), in early-stage natural or genetically induced immunoincompetence; (e) High fecundity and small size facilitate high-throughput drug and genetic screening at scale; (f) Diverse routes of drug administration are feasible (e.g., immersion, injection, oral), enabling flexible pharmacological studies; (g) Zebrafish models increasingly support personalized medicine pipelines by predicting patient-specific therapy responses; (h) As early-stage larvae are not legally classified as animals in many jurisdictions, the model allows for rapid and uncomplicated study initiation.

Despite its advantages, the zebrafish model has several limitations: (a) Adult zebrafish are pigmented unless using specific mutant strains, reducing imaging quality compared to embryos; (b) Tumor xenografts typically involve small cell numbers, which may limit the modeling of intratumoral heterogeneity and TME interactions; (c) Cross-species differences in signaling ligands, chemokines, and immune interactions may reduce translational fidelity; (d) The optimal temperature for zebrafish (28.5 ˚C) differs from the 37 ˚C optimal for mammals; thus, xenografting of human cells is typically performed at a compromise temperature of 32–34 ˚C; (e) Zebrafish lack certain mammalian organs (e.g., mammary glands), limiting modeling of some human cancers; (f) Limited data on zebrafish-specific pharmacokinetics (ADME/PK-PD) may necessitate follow-up validation in rodent models; (g) While adaptable for therapy testing, immunotherapy studies remain constrained due to immature immune system in larvae and species-specific immune barriers (though the development of a humanized model offers more possibilities to study hematopoietic cancers [195]); and (h) As adult zebrafish are often subject to animal protection laws, maintaining a zebrafish colony may be less cost-efficient and require additional approvals.

## Fruit fly (*Drosophila melanogaster*)

### General experimental approach and alternative setups

*Drosophila melanogaster* has served as a central model organism in developmental biology and cancer genetics for over a century. It offers a genetically tractable and cost-effective platform to study tumorigenesis at the organismal scale. As a non-vertebrate model, *Drosophila* aligns with the 3R principle—particularly Replacement and Reduction—while enabling detailed mechanistic studies of cancer biology [196, 197]. This includes the first links between phenotypes and genes and chromosomes in the famous Fly Room of Thomas Hunt Morgan [198] and the discovery of cancer-related pathways such as Hedgehog [24], Hippo [199], Notch [200], Wnt/wg [201], EGFR/Ras/MAPK, JAK/STAT, and PI3K/Akt/mTOR [202].

Experimental protocols typically begin with genetic crosses to combine tissue-specific drivers and oncogenic or RNAi constructs. Within days of adult emergence, flies develop visible tumor phenotypes—ranging from tissue hypertrophy to disseminated growth [203, 204]. These can be assessed macroscopically (e.g., bloating, reduced motility, lethality) or microscopically using fluorescent reporters and immunostaining. Environmental factors such as hypoxia, oxidative stress, hyperthermia, or dietary restriction can be modulated to study context-dependent tumor behavior [203].

The fly’s genetic toolkit enables the study of tumor initiation, progression, and therapy response [205], leveraging RNAi lines, CRISPR-Cas9, guideRNAs, and inducible transgene systems. These resources have allowed the modeling of diverse cancers, including colorectal, breast, glial, and hematologic malignancies (reviewed in [27, 203, 205–212]. In addition, spatial and temporal gene control permits the induction of tumors closely mimicking human cancers [203, 213, 214]. Indeed, tumor modeling in *Drosophila* typically relies on the binary GAL4/UAS expression system, which allows combined spatiotemporal control of (onco-)gene expression (e.g., RasV12) or tumor suppressor gene knockdown (e.g., p53, PTEN, APC) [215, 216] in specific tissues [217–219]. Here, intestinal stem cells (ISCs), neural precursors, imaginal discs, and hemocytes are commonly targeted [213, 220], while the Flippase/Flippase Recognition Target (FLP/FRT) or Mosaic Analysis with a Repressible Cell Marker (MARCM) systems enable clonal analysis of tumor growth and cell competition in a lineage-specific manner [221, 222].

Upon gene expression induction, flies develop neoplastic growth in epithelial or neural tissues. These tumor masses can even be serially transplanted into host flies to investigate invasion and metastasis [223, 224]. Recent advances further allow dissection of microenvironmental and inter-organ interactions, e.g., using dual expression systems (e.g., LexA/LexAop in parallel with GAL4/UAS) to manipulate tumor and stromal compartments independently [225, 226].

The short generation time (~ 10 days), low maintenance cost, and robust genetic tools make *Drosophila* uniquely suited for large-scale cancer biology experiments and mechanistic dissection of tumorigenic processes [196, 197].

### Analysis and experiment readouts

The *Drosophila* model offers countless methods to assess consequences of genetic mutations, but also studies of diet, age, irradiation, hypoxia, hyperthermia, oxidative stress, immune rejection, TME *et cetera* are possible as well as their use in combination (e.g., irradiation plus genetic/drug inhibition). The tractable anatomy and short lifespan of *Drosophila* facilitate high-throughput phenotyping and detailed analysis of tumorigenesis [227]. Tumor development can be macroscopically scored (e.g., abdominal distension, lethality) or microscopically evaluated via fluorescent reporters [228–230]. Tumor growth, proliferation, apoptosis, and differentiation are quantifiable using immunohistochemistry, reporter lines (e.g., Notch-, Wnt-, MAPK-activity sensors), and cell-type-specific markers [214, 231–233]. Invasion and metastasis studies leverage tracking of disseminating cells via fluorescent lineage markers [223, 224].

Fluorescently labeled transgenes under tissue-specific drivers allow *in vivo* imaging of tumor formation, clonal expansion, and disseminations [217]. Tumor cell proliferation can be tracked by phospho-histone H3 staining, while apoptosis is assessed using cleaved Caspase-3 or TUNEL assays [234]. Tissue architecture and differentiation states are further analyzed by immunohistochemistry for epithelial or lineage-specific markers [235].

Clonal and lineage-tracing tools such as MARCM [222], Flp-out [236], and ReDDM [237–239] can be used to address the effects of intrinsic and extrinsic factors on tumor behavior with spatiotemporal control of the onset of tracing and genetic manipulation [240]. Observations such as changes in cell fate in tumors of flies [241] complement and add functional knowledge to the worsened survival of patients with, e.g., CRC with a high percentage of enteroendocrine cells [242].

Functional readouts of signaling pathway activity are routinely performed using genetically encoded reporters. For example, Notch [243], Wnt/Wingless [244], Ras/MAPK [245, 246], and Jak/Stat [236] activity can be tracked using fluorescent transcriptional sensors, allowing quantitative assessment of pathway dynamics before and after tumor initiation or therapeutic intervention [247–249].

In recent years, *Drosophila* cancer models have been used to investigate tumor cell dissemination from primary tumours [250–252] involving processes and/or factors stimulating EMT in flies [237, 238]. Emerging methods such as FACS allow isolation of circulating tumor-like cells from whole-fly homogenates for downstream transcriptomic or proteomic analyses [73, 250, 251, 253]. Combined with transplantation assays, this supports the assessment of tumor invasiveness and metastatic potential *in vivo* [254, 255]. Consequently, new genetic approaches taking advantage of dual expression systems can be employed to assess and manipulate TME effects and organotropism *in vivo* [246, 256]. Together, these sophisticated methods allow in-depth investigation of early metastatic events with single-cell resolution and drug screens aiming to identify molecules inhibiting metastasis.

### Therapy modalities

*Drosophila* offers a versatile and high-throughput system for *in vivo* testing of anticancer therapies. Compounds are most commonly administered by blending them into fly food, ensuring systemic exposure with minimal technical effort and allowing for scalable screening [227, 257, 258]. Alternative application routes such as topical delivery and microinjection are also possible, yet are used less frequently due to throughput and practicality considerations [259].

Thanks to an almost 80% conservation of disease- and cancer-causing genes, drugs developed for human application often work in flies [202]. Studies from the Cagan lab set the cornerstone for more complex drug screening approaches, including repurposing of FDA-approved drugs not currently used in cancer therapy [260].

Therapeutic efficacy is assessed by measuring changes in survival rates, tumor burden, or molecular pathway activity. Researchers employ tissue-specific fluorescent reporters and immunostaining to visualize and quantify tumor growth and regression [251, 258]. Targeted inhibitors of conserved cancer pathways—such as MEK, PI3K, and mTOR—have shown efficacy in *Drosophila* models with oncogenic alterations present in many human tumors [217, 259, 261]. Quantitative imaging and the use of genetic pathway sensors (for example, for Notch, Ras/MAPK, or Wnt signaling) enable real-time monitoring of the effects of various treatments [251, 262].

Radiotherapy, while not a routine modality in *Drosophila* studies, can still be modeled using low-dose ionizing irradiation to explore DNA damage responses and mechanisms of radiosensitization [263–265]. However, *Drosophila* does not allow for the precise, clinically relevant application typical in mammalian models.

Due to the absence of adaptive immunity in flies, direct modeling of immunotherapies is not possible [266]. Nevertheless, interactions between tumors, stromal cells, and the innate immune system can be experimentally interrogated and provide insight into TME dynamics [267–270].

**Fig. 3:**
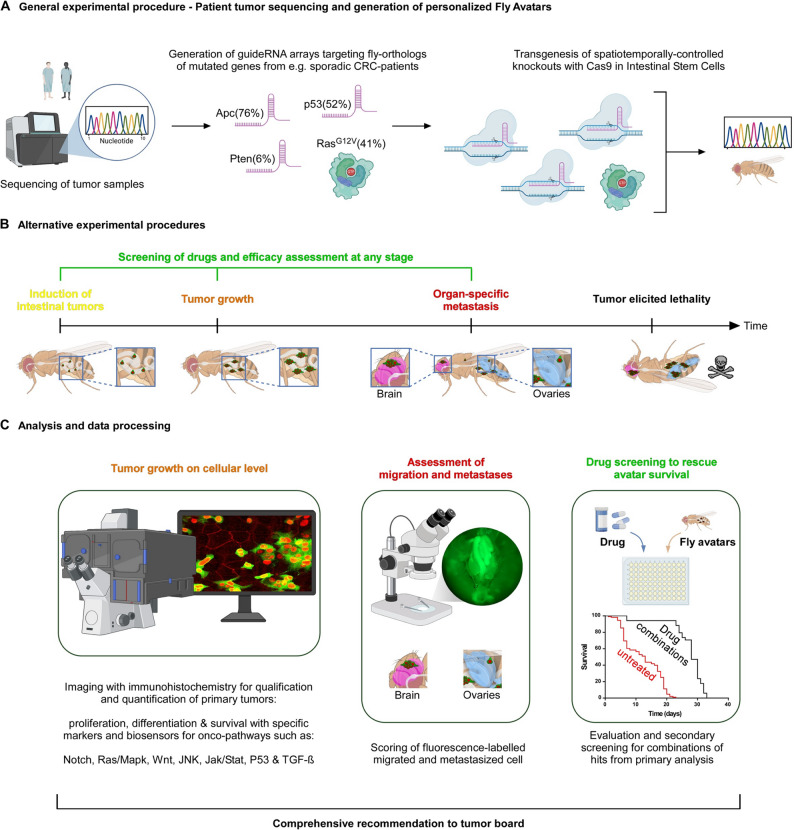
Overview of *Drosophila* model. **A**. Generation of personalized fly avatars after sequencing of tumor samples. CRC: colorectal cancer. **B**. Schematic representation showing a timeline for screening of drugs and efficacy assessment using fly avatars, starting with the induction of intestinal tumors in adult flies and proceeding with tumor growth, organ-specific metastasis, and finally tumor-elicited lethality. **C**. Analysis and data processing. Left: Analysis of tumor growth on cellular level by microscopic imaging of tumor-bearing intestines with fluorescent labeling of intestinal stem cells. Center: Assessment of migration and metastases of fluorescently labeled and traced tumor cells. Right: Drug screening to rescue avatar survival by high-throughput assays testing single drugs and drug combinations printed on microwell plates and CRC avatar flies. Effective drugs will be identified by their ability to prolong the survival of avatar flies and are further tested by their impact on tumor growth and metastasis formation in the other analyses. The combined results will contribute to recommendations for the tumor board. Created in BioRender (https://BioRender.com)

In summary, *Drosophila melanogaster* is highly suitable for early-phase screening of anticancer drugs and combination therapies. While not fully compatible with every clinical modality, this system offers a highly upscaleable, rapid, cost-effective, and ethically favorable platform for the *in vivo* assessment of therapeutic efficacy in genetically defined tumor models [227, 258, 261] (Fig. 3B).

### Patient-derived tumor models

An alternative to xenotransplantation performed in larger animals, *Drosophila* genetics allow the simulation of patient-specific cancer genotypes through the generation of “fly avatars.” These avatars are produced by introducing combinations of oncogenic mutations—identified from patient tumor sequencing—into specific tissues of the fly [205, 262, 271]. This system enables high-throughput drug screening, wherein disease-modeling flies are crossed with RNAi or CRISPR libraries and treated with candidate compounds delivered through food or topical application. Quantifiable phenotypic readouts—such as tumor size, signaling pathway activation, or organismal survival—allow for the rapid identification of effective, genotype-matched drug responses [272–274]. A first demonstration that drugs identified in flies can translate into clinical treatment of medullary thyroid carcinoma [275] paved the way for directed development of precise genetically complex fly models.

Technological advances have further refined this pipeline. Bangi et al. (2016) introduced multiplexed short-hairpin RNAi (shRNAi) arrays and semi-automated robotic screening, significantly accelerating the timeline of avatar creation and drug testing—an essential improvement for late-stage cancer patients [262]. Combining this approach with CRISPR-Cas9 technology, Zipper et al. (2022) developed a protocol involving scarless restriction enzyme strategies for multiplexed guide RNA array generation. This method improves editing efficiency, mitigates genetic compensation mechanisms, and reduces development time for complex avatars [276] (Fig. 3A).

Taken together, *Drosophila* avatars constitute a rapid, cost-efficient, and 3R-compliant platform for modeling complex patient-specific mutational landscapes and guiding targeted therapeutic development at scale.

### Advantages and limitations

The *Drosophila melanogaster* system offers several unique advantages for cancer modeling and drug discovery: (a) A powerful and flexible genetic toolkit—including GAL4/UAS, FLP/FRT, MARCM, RNAi, CRISPR, and dual expression systems—enables precise spatial and temporal control of gene expression and modeling of complex mutational landscapes; (b) Rapid generation time (~ 10 days) and low maintenance expenses allow for high-throughput, statistically robust screening with large biological replicates; (c) Fluorescent reporters and genetically encoded pathway sensors (e.g., for Notch, Wnt, Ras/MAPK, JAK/STAT) enable live tracking of tumor growth, clonal behavior, and therapy response at cellular resolution; (d) Fly avatars—genetically engineered to recapitulate patient-specific mutations—support personalized drug screening using multiplex RNAi/CRISPR approaches and semi-automated readouts; (e) Serial transplantation of fly tumors enables assessment of tumor cell propagation, invasiveness, and metastatic behavior *in vivo*; (f) Genetic and environmental manipulation targeting tumor or normal tissue enable investigation of inter-organ signaling and systemic effects such as cachexia; (g) Despite lacking an adaptive immune system, innate immunity and tumor-stromal interactions are accessible for mechanistic studies, (h) Maintenance of fly models requires minimal infrastructure and is generally inexpensive.

Nonetheless, several limitations must be considered: (a) As a non-vertebrate, *Drosophila* lacks certain anatomical structures (e.g., vasculature, bone marrow, lungs) and physiological systems (e.g., adaptive immunity), limiting its utility for modeling particular organ-specific pathologies, interaction of tumor cells and vasculature, and immunotherapies; (b) Drug pharmacokinetics and metabolism differ from humans; (c) Radiotherapy is only feasible using low-dose, non-targeted irradiation; (d) While fly avatars model mutational complexity well, they do not include patient-derived cells or native human TME; (e) Tumor histology and tissue architecture in flies differ from mammals, which may limit modeling of some tumor types or stromal interactions; (f) The small body size restricts longitudinal monitoring of systemic disease processes like tumor-induced immunosuppression; (g) Some conserved signaling pathways show context-dependent functional divergence, requiring cautious extrapolation to human biology.

In summary, *Drosophila* represents a fast, cost-effective, and ethically favorable *in vivo* model for genetic dissection of tumorigenesis and early-phase drug screening (Fig. 3C). While it lacks several mammalian features essential for translational cancer research, its unparalleled genetic tractability and screening capacity make it a powerful component in the preclinical modeling pipeline. Undoubtedly, the future of cancer research in the fly is bright: Might it be the arrival of prime editing allowing oncogenic transformation *in vivo* [277] or CAR-M (RaceCAR macrophages) cancer immunotherapy [270], the integration of newest discoveries into well-described model organisms will continue to revolutionize the field of 3R-compliant cancer modeling.

## Caenorhabditis elegans

### General experimental approach and alternative setups

The nematode *C. elegans* is a metazoan model organism first introduced by Sydney Brenner as a platform to study developmental biology and cellular processes *in vivo* [233, 278]. Its invariant and deterministic cell fates allow the tracking of each individual somatic cell during embryogenesis and larval development, from their origin until their physiological selective elimination [279, 280]. Notably, cell death defective (ced) *C. elegans* mutants enabled the discovery of programmed cell death as a genetically regulated phenomenon [281]. Indeed, Horvitz and colleagues identified the genes CED-3, CED-4, and CED-9 as core components of apoptosis, which later revealed functional homology to the mammalian caspase cascade and BCL2 pathway—establishing apoptosis as a conserved tumor suppressor mechanism [281, 282].

**Fig. 4:**
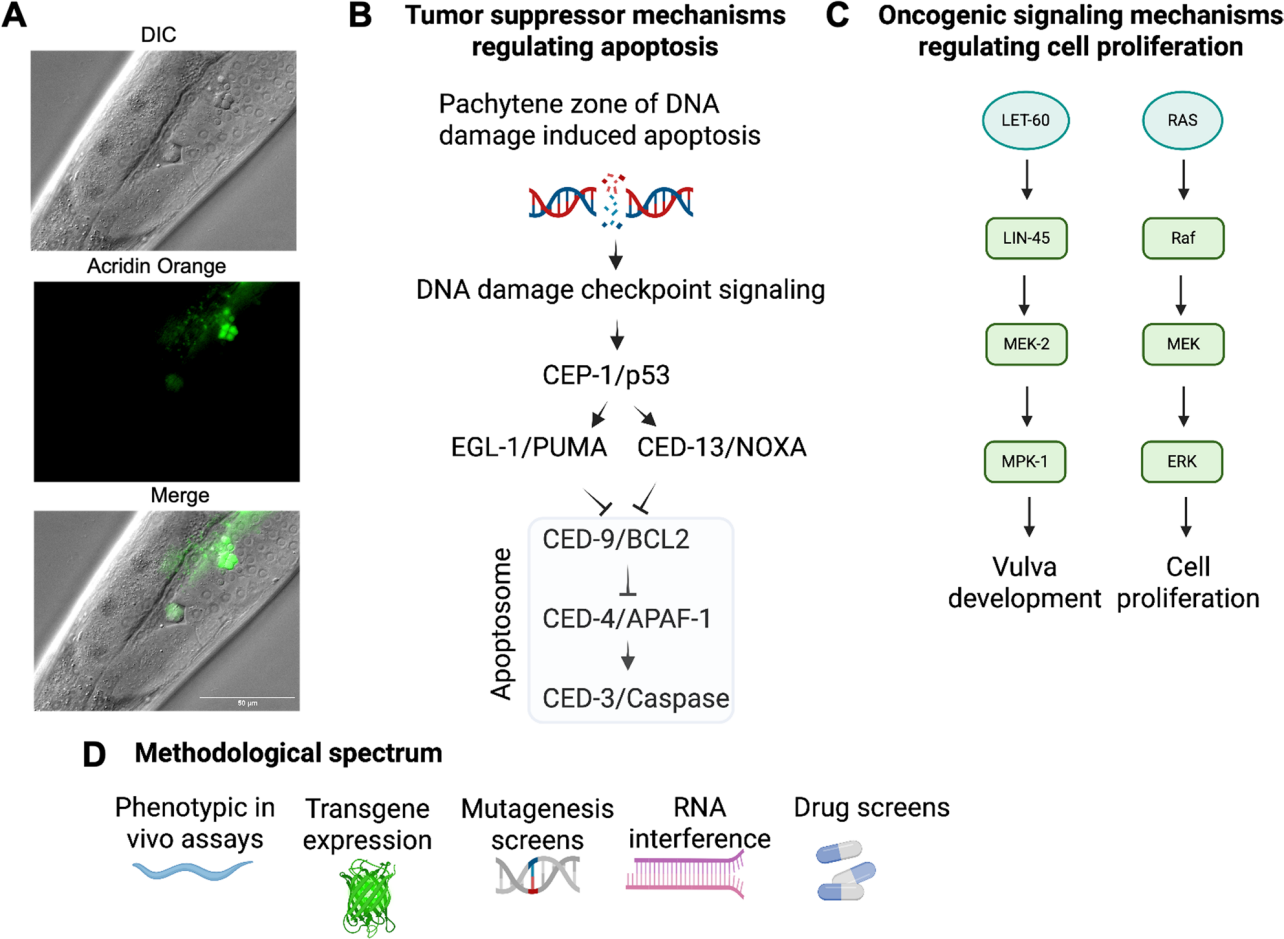
Overview of *C. elegans* model. **A**. In the late meiotic pachytene zone, apoptotic corpses are formed that can be visualized in differential interference contrast (DIC) microscopy and stained with Acridine Orange (AO). **B**. DNA damage in late meiotic pachytene cells triggers highly conserved DNA damage checkpoint signaling that activates the tumor suppressor CEP-1/p53, which transcriptionally induces the BH3-only domain proteins EGL-1 and CED-13. These PUMA and NOXA homologs inactivate the BCL-2 homolog CED-9, which then releases CED-4 to trigger the activation of the caspase CED-3, thus executing apoptosis. **C**. The proto-oncogenic RAS signaling pathway regulates cell divisions during the development of the vulva, and hyperactivation leads to extra cell division rounds, resulting in the formation of multiple vulvas. **D**. A wide range of methodologies allows the in vivo analysis of the consequences of mutations in conserved tumor suppressors or oncogenes in distinct tissue types, developmental stages, and aging. Transgenes can be expressed with fluorescent markers or other tags, mutations can be generated by gene editing or mutagenesis, and genes can also be knocked down by RNAi. The effects of small molecules such as chemotherapeutic drugs can be assessed upon feeding the animals on plates supplemented with such molecules or exposing them in liquid culture to drugs. A generated by Dr. Christina Efraimoglou, B-D created in BioRender (https://BioRender.com)

While the nematode doesn’t develop cancer largely due to the early developmental terminal differentiation of the somatic cells, that in the adult are devoid of any cell division potential, the germline can develop tumors, providing a dynamic model for genome stability, DNA damage response (DDR), and oncogenic signaling [283, 284]. Upon DNA damage, the *C. elegans* p53-like CEP-1 protein induces pro-apoptotic factors EGL-1 and CED-13, which inactivate CED-9 to trigger cell death (Fig. 4A) [285, 286]. As p53 is the single most frequently mutated gene in human cancers [287], the availability of a homolog in a powerful genetic system enabled the discovery of novel regulatory mechanisms. Based on discoveries in the nematode model, conserved layers of p53 regulation were uncovered, including translational repression by GLD-1 and protein turnover via SCF and CYLD complexes [288–293].

Conserved oncogenic signaling pathways can be interrogated in *C. elegans* using genetically tractable models with well-defined phenotypic outputs. For example, mutations in RAS/MAPK pathway genes such as *let-60* (RAS homolog) and *mpk-1* (ERK/MAPK) result in a multivulva phenotype, providing a direct and quantifiable readout of RAS/MAPK pathway activation *in vivo*(Fig. 4B) [294–296]. Similarly, loss-of-function mutations in *gld-1*, a translational repressor required for meiotic entry, induce germline tumors due to mitotic re-entry of undifferentiated cells [297, 298]. These models facilitate the functional analysis of oncogene activation, cell fate disruption, and proliferative transformation in a genetically defined system. Modern genetic tools—including chemical mutagenesis, RNAi knockdown, and CRISPR/Cas9 genome editing—enable the generation of loss- or gain-of-function alleles, reporter strains, and targeted knock-ins (Fig. 4C) [299–301].

Gene mutations can either be introduced stochastically or through targeted approaches. Historically, chemical mutagenesis, for instance, through N-ethyl-N-nitrosourea (ENU) exposure, has been used to generate strains carrying mutant alleles, which are then phenotypically characterized and the underlying mutation identified. Since the advent of whole genome sequencing (WGS) approaches, the identification of mutant alleles has been greatly improved [302]. A major step towards studying gene function was the implementation of RNA interference approaches that could in principle allow knocking down any of the roughly 20,000 nematode genes [303, 304]. In recent years, CRISPR/Cas9 gene editing methods were also advanced in *C. elegans* and are routinely applied to generate mutant alleles, including loss-of-function and gain-of-function alleles, as well as knock-in constructs ranging from engineered promoters to various types of tags [305]. Notably, *C. elegans* has recently been applied in a translational setting as a functional diagnostic tool, where chemotaxis-based N-NOSE testing enabled prediction of chemotherapy response in esophageal cancer patients, underscoring its potential utility in treatment stratification [306].

Together, these features establish *C. elegans* as a powerful, 3R-compliant model for dissecting conserved cancer-related mechanisms such as apoptosis, DNA damage signaling, and oncogenic transformation at single-cell resolution in a whole-organism context.

### Analysis and experiment readouts

*C. elegans* provides a powerful and genetically accessible platform for *in vivo* analysis of cancer-relevant processes such as apoptosis, DNA damage response, and oncogenic signaling (Fig. 4). Its optical transparency and invariant cell lineage facilitate dynamic tracking of molecular and cellular changes across development and under stress conditions.

Fluorescent reporters, introduced through germline microinjection, allow dynamic tracking of gene expression and protein localization in live animals [307]. These constructs can report transcriptional states, monitor subcellular protein distribution, or pathway activation in response to physiological or genotoxic stimuli [308]. The optical transparency of *C. elegans* supports real-time visualization of cellular processes such as mitosis, protein dynamics, and apoptosis [280, 309]. Apoptotic events can be identified via their characteristic refractile morphology under DIC microscopy or using fluorescent markers such as CED-1::GFP or Acridine Orange staining [283, 310, 311]. Developmental apoptosis is commonly quantified by scoring persistent ‘undead’ cells in ced mutants [282, 312], while DNA damage-induced germline apoptosis serves as a robust functional readout of DNA damage response (DDR) gene activity [284, 289]. For instance, defects in global-genome nucleotide excision repair (GG-NER) that in humans lead to exquisite hypersensitivity of UV-induced skin cancer lead to genome instability in germ cells of *C. elegans* [313–315].

Assays involving exposure to genotoxic agents like ionizing radiation (IR) or ultraviolet (UV) light are commonly used to assess genome integrity. IR mainly affects proliferating germ cells and early embryos, while UV exposure induces DNA damage and growth arrest in somatic tissues [316, 317]. These assays facilitate the dissection of conserved DNA repair pathways and allow functional interrogation of genome maintenance mechanisms [285, 318].

Oncogenic signaling can be monitored using genetically defined phenotypic outputs. As mentioned above, constitutive RAS/MAPK activation results in a multivulva phenotype that provides a direct and quantifiable endpoint [294]. In the germline, aberrant ERK signaling disrupts oocyte differentiation, and loss of *gld-1* leads to tumor-like mitotic overproliferation [297, 319]. These phenotypes serve as reliable indicators of disrupted differentiation and proliferative transformation.

Altogether, these analytical tools make *C. elegans* a highly informative and scalable model for functional studies of cancer-relevant genes, processes, and pathways *in vivo*.

### Therapy modalities

*C. elegans* can not only be used to obtain insights into basic cancer biology, but it could also aid in better understanding therapeutic targets, modes of action, and side effects of both novel and established pharmaceuticals. Drugs, including chemotherapeutics, can be easily administered to *C. elegans* by incorporating the compounds into the nematode’s growth environment through agar plate supplementation, where for instance common chemotherapeutics such as cisplatin and doxorubicin have been assayed [320, 321]. In addition, drugs may be administered via bacterial diet incorporation, where they are added to the bacterial feeding source prior to seeding the agar plates. An advantage of this approach is the possibility to study the contribution of the bacteria’s metabolism to the preprocessing of certain drugs, which can shed light on the effect of the human bacterial microbiota on cancer drug efficacy—an emerging concern in human cancer therapy [322]. Either of these methods provides a straightforward, reproducible, and scalable drug administration system for *C. elegans*.

In terms of drug discovery, the ease to produce large populations of worms simplifies the implementation of high-throughput approaches to screen small-molecule libraries [323]. Provided that drugs may be absorbed directly through the nematode’s cuticles, it is worth noting that several *C. elegans* strains carrying mutations affecting cuticle integrity have been reported to have increased drug absorption [324]. Using these strains with increased cuticle permeability in drug screens may reduce potential false-negative hits due to insufficient doses reaching the target cells. Overall, the use of *C. elegans* in the context of drug testing presents clear advantages in terms of cost, data robustness, and low amounts of required chemicals, which may facilitate the testing at early stages of compound development.

### Advantages and limitations

*C. elegans* has facilitated the discovery of biological phenomena, as evidenced by the multitude of Nobel Prizes awarded to worm researchers. Taken together, *C. elegans* has provided important mechanistic insight into the function of processes that are causally linked to cancer development while providing distinct advantages: (a) It provides a simple animal model with distinct cell and tissue types; (b) The life cycle is short, and within three days after birth the reproductive age is reached and each hermaphrodite can produce 300 offspring in a matter of days; (c) Over 70% of the cancer-related genes and pathways are highly conserved and can be studied in the worm [325]; (d) Even highly complex biologically phenomena such as the organism wide systemic responses to genome instability have been identified in this system; (e) Inheritable consequences of DNA damage such as the formation of de novo structural variants can be followed through generations and the relative contribution of DNA repair and epigenetic mechanisms can be investigated; and (f) It provides a suitable platform not only for genetic screening but also for *in vivo* functional investigation of mechanisms in development, adulthood and aging.

Conversely, the simplicity and evolutionary distance of *C. elegans* pose limitations on studying complex human diseases such as cancer. While the mechanisms of genome stability and the DDR including the most important tumor suppressors such as p53 and PTEN as well as oncogenes such as RAS and MAPK signaling cascades are conserved, the physiology of a cancer, with its different cell types, metastasis, and interactions with the immune system clearly require higher experimental animal models. Despite these limitations, the worm keeps its promise of a simple metazoan 3R-compliant model that allows the understanding of fundamental and novel phenomena at the whole-organism scale.

## Guidelines for choosing a 3R-compliant alternative animal model

Despite the many advances in developing improved and more sophisticated *in silico* and *in vitro* models, ranging from the implementation of more physiologically relevant media and growth patterns (e.g., 3D, organoids) to AI-guided molecular predictions, animal models are still indispensable to characterize and recapitulate the complex nature of cancer. Indeed, mammalian models—predominantly rodents—have played a crucial role in elucidating the causes and mechanisms of human cancers, helping characterize phenotypic hallmarks, and enabling the preclinical evaluation of existing and experimental drugs [326, 327]. The advantages and inherent limitations of murine cancer models, particularly regarding translational relevance and species-specific differences, have been thoroughly reviewed elsewhere [328, 329]. Importantly, questions requiring long-term tumor evolution, complex pharmacokinetics, intact adaptive immunity, or higher-order neuroendocrine regulation may still necessitate rodent—and in specific cases primate—models to capture organism-level physiology [328, 330, 331]. Nonetheless, the disadvantages of rodent models as well as the necessary implementation of the 3R principle has encouraged the use of simpler model organisms to not only reduce ethical concerns but also promote efficient experimentation while yielding clinically relevant results at lower costs and faster experimental timelines.

**Fig. 5:**
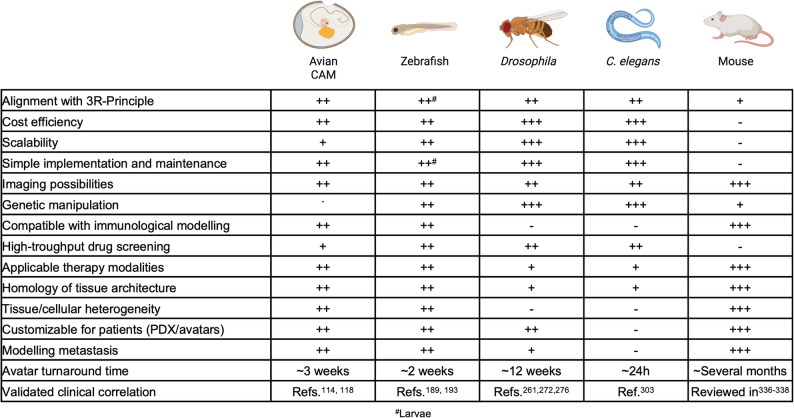
Comparative experimental characteristics of different animal cancer models. Overview of advantages (+) and disadvantages (-) of the avian CAM, zebrafish, *Drosophila*, *C. elegans*, and mouse models in cancer research. Created in BioRender (https://BioRender.com)

Selecting an appropriate animal model depends on the biological question (Fig. 5): For dissecting conserved mechanisms such as apoptosis or genome maintenance, invertebrate models like *C. elegans* and *Drosophila* offer powerful genetic tools, fast generation times, and single-cell resolution. *C. elegans* is particularly suited for studying DNA repair, genome integrity, and apoptosis at high cellular resolution, while *Drosophila* offers precise options for modeling complex tumor genetics, tissue-specific transformation, and drug screening. When tissue organization, tumor heterogeneity, or invasion are of interest, vertebrate systems like zebrafish or the avian CAM model are more suitable due to their complex physiology and compatibility with xenotransplantation. Zebrafish provide a genetically tractable vertebrate system with whole-organism imaging and transgenesis, whereas the avian CAM model excels in short-term, medium-throughput xenograft assays with minimal required infrastructure. Importantly, it should be noted that the degree of genetic conservation described for each model reflects the specific experimental needs and constraints of the respective research communities rather than a uniform measure of proximity to human biology.

For large-scale drug or genetic screens, models with short life cycles and simple husbandry—such as *C. elegans*, *Drosophila*, and zebrafish embryos—enable high-throughput experimentation. If the research involves microbiota host-tumor interactions, or pharmacokinetics, systems with intact physiology like zebrafish or CAM*—*or leveraging bacteria in combination with *C. elegans—*provide greater translational relevance. In practice, the complementary use of models often yields the most robust insight across molecular, cellular, and organismal levels.

Beyond mechanistic suitability, an important selection criterion is predictive validity—i.e., whether responses observed in the model correlate with patient outcomes. Importantly, evidence levels differ: some platforms enable patient-matched therapy testing (same regimen tested in the model), whereas others primarily infer putative sensitivities from genetic alterations or pathway dependencies. Translational validity, evidence levels, turnaround times, and key constraints of the discussed model systems are covered in dedicated reviews for interested readers (avian embryo [97, 332]; zebrafish [181, 183]; Drosophila [227, 229]; C. elegans [333, 334]; rodents [335–337]; different *in vitro* and *in vivo* model systems [338, 339]).

Overall, emerging tools in genome editing, single-cell analysis, and live imaging are unlocking new potential in 3R-aligned models, broadening their impact from basic discovery to clinical application. Leveraging each model’s unique features will foster an efficient and 3R-compliant future for cancer research.

## Data Availability

No datasets were generated or analysed during the current study.
